# High-Performance Work System, Strategic Flexibility, and Organizational Performance—The Moderating Role of Social Networks

**DOI:** 10.3389/fpsyg.2021.670132

**Published:** 2021-11-15

**Authors:** Yizhi Wang, Yi Cao, Nan Xi, Huitian Chen

**Affiliations:** ^1^The Institute of Public Administration and Human Resources, Development Research Center of the State Council, Beijing, China; ^2^School of Psychological and Cognitive Sciences, Peking University, Beijing, China; ^3^School of Labor Economics, Capital University of Economics and Business, Beijing, China; ^4^Middlebury College, Middlebury, VT, United States

**Keywords:** dynamic capability theory, high-performance work system, organizational performance, strategic flexibility, social network

## Abstract

Based on the dynamic capability theory, this research investigated the effect of a high-performance work system on organizational performance, the mediating role of strategic flexibility, and the moderating role of an enterprise’s social network in this relationship. A total of 214 middle and senior managers from 58 Chinese enterprises were invited to participate in this research. The results showed that the high-performance work system is positively correlated with organizational performance and such correlation is partially mediated by strategic flexibility. Results found that the social network of an enterprise negatively moderated the relationship between a high-performance work system and strategic flexibility. However, the social network did not moderate the mediating role of strategic flexibility in high-performance work systems (HPWS) and organizational performance.

## Introduction

Enterprises are facing increasing uncertainties of the environment and market as a result of dynamic competition in recent years. At the same time, strategic flexibility has become a major approach in allowing enterprises to maintain competitive advantages in varied situations (Cao et al., 2018). Although many researchers have demonstrated the positive relationship between strategic flexibility and organizational competitive advantages or organizational performance (Han and Gao, 2017; Lin et al., 2017), the antecedent variables of strategic flexibility, especially its relationship with High-performance Work Systems (HPWS) is rarely explored. Accordingly, whether HPWS can improve organizational performance through strategic flexibility remains controversial. HPWS refers to a series of different but interrelated human resource practices implemented by enterprises, which aim to improve individual and organizational performance by improving employees’ abilities, attitudes, and motivations (Takeuchi et al., 2009; Miao et al., 2013).

Previously, a large body of research had found that HPWS are positively related to organizational performance, including decreasing employee turnover rate (Arthur, 1994; Guthrie, 2001), increasing employee satisfaction (Wright and Snell, 1991), labor productivity, and business performance (Huselid, 1995). However, some research in recent years has also challenged these conclusions because of environmental uncertainty. For example, Shin and Konrad (2017) found that there is a reverse causal relationship between HPWS and organizational performance. Researchers in the field of institutional theory pointed out that with the people-oriented concept deeply rooted in the mind of employees, the labor union may hinder the positive relationship between HPWS and organizational performance (Jackson et al., 2014). Accordingly, Wright et al. (2005) and Guest et al. (2013) also suggested that the positive relationship between HPWS and organizational performance will disappear after controlling the influence of past performance. We proposed that the contradictory conclusions mentioned above may be caused by: (1) the fact that previous research has not fully considered the impact of the dynamic environment when exploring the mechanism of HPWS on organizational performance (Batt, 2002; Jiang et al., 2013; Miao et al., 2013); and (2) the impact of boundary conditions between HPWS and outcome variables has not been fully considered.

Based on dynamic capability theory (Teece et al., 2015), strategic flexibility can help enterprises prepare to adapt to changes in the environment (Sanchez, 1995, 1997). Xing et al. (2015) have suggested that enterprises can implement HPWS in an uncertain environment to allocate human resources rationally, which could further improve strategic adjustment and contribute to organizational performance. Recently, strategic flexibility has been increasingly used to explain the relationship between the top management team and performance in the dynamic environment (Wang, 2015; Li et al., 2016). On the contrary, the perspective of strategic human resource management rarely considered strategic flexibility as an organizational capability. The dynamic capability theory indicates that strategic flexibility, an organizational capability in a dynamic environment (Han and Gao, 2017), involves the ability to adapt to environmental changes or actively change the environment (Nadkarni and Herrmann, 2010). This ability is the core competitive advantage of an enterprise. Accordingly, HPWS are considered to be the key approaches to improving the organizational capabilities of enterprises. In a dynamic environment, HPWS can enable enterprises to respond to changes in the external environment by improving strategic flexibility and further improving organizational performance. Unfortunately, there has been research on the relationship between different HPWS, strategic flexibility, and organizational performance.

The implementation effect of HPWS is not universal (Liang and Cui, 2012) and can be affected by other factors in society (Miao et al., 2013), especially in the “Guanxi”(a word for describing general relationship) society in China. The idea that “Guanxi is productivity” or that “Guanxi is a resource” can be applied to not only individuals but also enterprises. Thus, the present research put the social network into the model from the perspective of sociology. The social network of an enterprise refers to a set of stable resources and interaction norms formed by relationships and interactions between enterprises. The broader the social networks of the enterprise, the higher resource richness, and network embeddedness. Such richness and embeddedness can bring better strategic adjustment ability and be able to find higher potential market opportunities (Casciaro et al., 2015). However, such resources and advantages do not stem from management capabilities but depend more on their inherent position in social networks (Ren and Rui, 2014). Conversely, for enterprises with a limited social network, their resources and network embeddedness will also be low. In other words, for such enterprises, their ability to find potential market opportunities and make strategic adjustments needs to rely on its own management capabilities (human resource management system). Therefore, enterprises with rich social network resources will rely less on their HPWS to obtain strategic flexibility. In conclusion, the capability of social networks and the capability of human resource management may conflict with each other in improving strategic flexibility. Thus, social networks may moderate the relationship between HPWS and strategic flexibility.

In summary, this research examined the role of strategic flexibility between HPWS, organizational performance, and the moderating role of social networks in this model from the perspective of sociology, the result of this research may give a theoretical reference for the management of enterprises.

## Theoretical Background and Hypotheses

### High-Performance Work System

The research on HPWS is based on the idea that a human resource management system is positively related to the improvement of organizational performance. Although the specific practices that should be included in HPWS are still controversial, such an expectation is supported by other researchers (Miao et al., 2013; Jackson et al., 2014). Huselid et al. (1997) pointed out that HPWS refers to “designing and implementing a series of internally coordinated human resource policies and practices to ensure the improvement of human capital (employees’ knowledge, skills, and abilities) to ultimately achieve the business goals.” Miao et al. (2016) also claimed that the practices of HPWS should be complementary and synergistic. Additionally, compared with single human resource practices, the bundle of human resource practices is more effective.

The differences between China and other countries in terms of social and economic development mean there are differences in the choice of HPWS. Countries in Europe and the US, have generally adapted commitment-oriented HPWS. However, an inconsistent transition from industrialization to scientific management has meant that Chinese enterprises place more emphasis on the combination of “commitment-oriented” and “control-oriented” HPWS (Miao et al., 2013). It should also be noted that, with the overall improvement of the quality of Chinese employees, the proportion of control-oriented human resource practices in HPWS has gradually decreased. Accordingly, in this research HPWS is based on commitment-oriented human resource practices, supplemented by control-oriented human resource practices. The commitment-oriented human resource practices are composed of recruitment, training, employee participation, internal market, information sharing, and salary management. The control-oriented human resource practices are composed of assessment and discipline management.

### High-Performance Work System and Organizational Performance

The relationship between HPWS and organizational performance has always been an important topic in research on strategic human resource management. Theories supporting the relationship between the two tend to focus on resource-based views (Barney, 1991), behavioral perspectives (Jackson et al., 2010), human capital theory (Becker, 1964), AMO theory (Appelbaum, 2000), social exchange theory (Blau, 1964), and institutional theory (Meyer and Rowan, 1977), with the resource-based theory the most used. The resource-based view claims that the resources that help enterprises maintain competitive advantages are the most heterogeneous ones, especially those valuable and scarce (Jackson et al., 2014; Saridakis et al., 2017), such as dedicated human resources. The enterprises that invest in those resources through the implementation of HPWS will improve the knowledge, skills, and abilities of employees. Such improvement can ultimately improve organizational performance (Ployhart and Moliterno, 2011). Moreover, in the dynamic environment, dynamic capability theory can be a good supplement to the resource-based view (Lin and Zhao, 2013). It contributes to a better explanation of the relationship between HPWS and organizational performance. Dynamic capability theory emphasized that enterprises need to have the ability to integrate, construct, and reconfigure internal and external resources quickly to adapt to a rapidly changing environment (Teece et al., 2015). This theory assumed enterprises with higher dynamic capabilities have more competitive advantages under the dynamic environment. We propose that HPWS can improve the effectiveness of enterprises in a dynamic environment. For example, team building, and flexible authorization have the function of reconfiguring and constructing internal and external resources. Policies such as encouraging information sharing are more conducive to the effective development and implementation of new opportunities by enterprises (Cao et al., 2018). The disciplines of the enterprise need to be followed strictly when facing key events so that business opportunities will not be missed. At the same time, incentives such as salary management can improve employees’ motivation and enable them to adapt to environmental changes more proactively. Therefore, HPWS can help enterprises to create, expand, and adjust their human resources strategically, which ultimately affects organizational performance.

*Hypothesis 1: HPWS is positively related to organizational performance*

### High-Performance Work System and Strategic Flexibility

Research on strategic flexibility stems from technological progress, intensified global market competition, the rise of learning organizations, and the emergence of new business models. Accordingly, to cope with environmental uncertainty, technological development, and market changes, enterprises need to improve their strategic flexibility. The concept of strategic flexibility was proposed by Sanchez (1995, 1997) and divided into resource flexibility and coordination flexibility. Previous research had pointed out that in a dynamic competitive environment, the resources of enterprises can easily become rigid and unable to adapt to the environment. Such changes may lead to the ultimate loss of inherent value especially for intellectual capital that is updating fast (Ketkar and Sett, 2009). The available value will be reduced greatly if not updated. Accordingly, enterprises need to continuously increase the flexibility of this type of resource for responding to changes (Barney, 1991). In addition, enterprises also need to have the ability to allocate resources reasonably and flexibly, especially for problems like poor process communication and opaque information sharing in the management process. Besides, coordination flexibility is also critical for enterprises to maintain competitive advantages. Based on the research of Evans (1991) and Sanchez (1995, 1997), present research defines strategic flexibility as “in response to the dramatic changes in the dynamic environment, enterprises use flexible and effective management to identify markets, adjust strategies, allocate resources, and obtain competition advantages.” Strategic flexibility can enable enterprises to obtain competitive advantages in the dynamic competitive environment, which motivates us to explore how enterprises obtain strategic flexibility (Martín et al., 2008). From the perspective of human resource management, scholars in the field of strategic human resource management have suggested that HPWS are an effective way of improving strategic flexibility (Sparrow, 1998).

Previously, researchers have explored the impact of HPWS on strategic flexibility from the perspective of management practices based on dynamic capability theory (Wright and Snell, 1998; Evans and Davis, 2005). For example, the practices of extensive training, internal market, and recruitment in HPWS can improve the learning atmosphere of employees and enable the enterprise to train employees to learn skills needed in the future (Martín et al., 2008). Thus, the enterprises’ ability to discover market opportunities will also be improved (Pavlou and El Sawy, 2011). Human resource practices such as rotation exchanges and career development can help employees expand their horizons, improve self-awareness, and determine what knowledge and abilities need to be improved and rebuilt, help them to respond to changes in the complex environment (Parker and Axtell, 2001). These practices can essentially increase the stock of employees’ intellectual capital, hence improving resource flexibility. In addition, the core of flexible authorization is to reintegrate the internal processes and architecture of the enterprise. Such reintegration can remove the information communication barriers brought by the previous bureaucracy, and “allow employees who can hear the gunfire to make decisions.” By doing so, the enterprise can make better decisions and quickly respond to changes in the external environment (Cao et al., 2018). Moreover, the internal information sharing mechanism implemented by the enterprise can promote the large-scale flow of information and resources within the enterprise, which can help the enterprise to identify external markets and opportunities continuously and quickly (Evans and Davis, 2005; Pavlou and El Sawy, 2006). These practices enhance coordination flexibility.

*Hypothesis 2: HPWS is positively related to strategic flexibility*

### Strategic Flexibility and Organizational Performance

Enterprises with higher strategic flexibility are more capable of responding to changes and challenges brought by uncertain environments in the dynamic competition (Cao et al., 2018) and improving organizational performance. Specifically, enterprise with resource flexibility can rely on its rich intellectual capital to make more strategic choices. As the old saying goes “Knowledge is power,” which means the richer the employee’s intellectual capital is, the more likely it can help the enterprise in the shortest time to realize the transformation of market strategy at the minimum cost, thereby reducing its market risks, enhancing its environmental adaptability, all of which will improve organizational performance. Furthermore, talents with multiple abilities and skills are more likely to deal with unknown challenges and seize fleeting strategic opportunities in a complex environment, thereby increasing the probability of an enterprise being a successful enterprise. From another perspective, enterprises with coordination flexibility are more likely to reintegrate and allocate resources in a dynamic competition in a timely manner to maximize the effectiveness of resources. Coordination flexibility not only saves the enterprise’s existing operating costs, but also improves the frequency of the enterprise’s new products, timely response to market competition, and improves organizational performance.

In addition, some research also pointed out that strategic flexibility has a significantly positive impact on organizational performance. For example, Li et al. (2016) took 350 Chinese enterprises as an example and found that strategic flexibility has a positive impact on organizational performance. Lin and Zhao (2013) suggested that strategic flexibility is a key element for enterprises to gain a competitive advantage in the twenty first century. In summary, strategic flexibility can help enterprises in quickly identifying and adapting to the ever-changing external environment, and bring more strategic opportunities, which can improve organizational performance.

*Hypothesis 3: Strategic flexibility is positively related to organizational performance*

### High-Performance Work System, Strategic Flexibility, and Organizational Performance

Previous research on the mediating mechanism between HPWS and organizational performance is often based on static resource-based views, using employee attitudes or behaviors as a mediating mechanism (Lin and Zhao, 2013). However, dynamic capability theory indicates that the resource-based view is a static theory, and it is difficult to explain the source of enterprises’ competitive advantage in eras of rapid change (Barney, 2001; Cao et al., 2018); however, dynamic capability theory can supplement this deficiency. Thus, the present research is based on dynamic capability theory to clarify the mechanism that how HPWS influence organizational performance through strategic flexibility.

Strategic flexibility is characterized by dynamic capabilities, specifically the ability to integrate and restructure resources to adapt to environmental changes (Batt, 2002). Research on strategic flexibility is based on dynamic capability theory, which suggests that the dynamic ability of an enterprise responding to the changing environment is the basis for maintaining its competitive advantage while existing resources and capabilities can only bring temporary competitive advantages to the enterprise (Sanchez, 1997). Strategic flexibility is essentially a dynamic capability of maintaining enterprise competitive advantages (Amit and Schoemaker, 1993) and implementing new strategic opportunities (March, 1991), which is based on continuously improving resource flexibility and resource use efficiency. Even though early research was based on the resource-based view, studies were already aware of the importance of dynamic capabilities (Wright et al., 2001). Moreover, they also indicate out that strategic human resource management can increase the enterprises’ intellectual capital stock to increase strategic flexibility, and thereby enhance the competitive advantage. Subsequent research was further based on the dynamic capability theory, using strategic flexibility as a mediating mechanism and found that strategic human resource management can reconfigure internal and external resources and capabilities to generate new knowledge and capabilities by discovering and seeking internal and external opportunities, thereby creating the competitive advantage (Becker and Huselid, 2006; Beltrán-Martín et al., 2008; Lin and Zhao, 2013).

From the perspective of HPWS, the present research suggests that by using a series of human resource practices, enterprises can continuously improve the knowledge and skills of employees, and increase the stock and flow of corporate intellectual capital. By doing so, the obstacles of communication and coordination can also be eliminated to a certain extent, and the flow of corporate knowledge and information will increase. Ultimately, the enterprises’ ability to respond to changes in the external environment will be enhanced and affect organizational performance.

*Hypothesis 4: Strategic flexibility mediates the relationship between HPWS and organizational performance*

### The Moderating Role of Social Networks

The importance of the social context and social networks had been gradually recognized by scholars (Granovetter, 1985; Nahapiet and Ghoshal, 2000). The term social network in this research refers to the organizational-level social network (Ahuja, 2000). It is a collection of stable resources and interaction norms formed by the relationships and interactions between different enterprises for obtaining and maintaining competitive advantages.

Even though HPWS can affect strategic flexibility, the effect can nevertheless vary in different social situations (Granovetter, 1985; Arenius and Clercq, 2005). Social network theory indicates that the social network presents a pyramid-like structure: the closer the position to the top of the tower is, the fewer the number of occupants will be, but the richer the resources they can master. Accordingly, the more important the position in the network is, occupants in this position can have a wider view of the entire network and easier access to the most valuable resources (Lin, 1999). The huge network itself brings enterprises the opportunity and ability to quickly identify the market opportunities and make strategic adjustments. In addition, if the network strength of the enterprise is high, there will be a high exchange of resources between enterprises, which often brings high proprietary capital to the enterprise. Thus, a strong relationship between each other (Granovetter, 1973) will be established and the trust between enterprises can be enhanced (Teece, 2009), which makes it easy to form a consistent shared mental model, values, and strategic goals (Evans and Davis, 2005). Thus, those enterprises can realize the flow of resources at a lower cost. When the market changes, enterprises can share risks, information and exchange resource quickly (Gargiulo and Benassi, 2000), flexibly adjust strategies, and jointly respond to market changes. Based on these benefits that social network brings to strategic flexibility, we can infer that if an enterprise has a high-level social network, and the positive effect of HPWS on strategic flexibility would decrease. Moreover, when the centrality of the enterprise in the social network is more important, it would occupy more structural holes in the social network, which can allow the enterprise to easily obtain valuable, non-redundant, and high-quality information for increasing strategic flexibility. In this context, no matter how effective the implementation of HPWS is, enterprises can obtain strategic flexibility not only on HPWS but also the rich resources and advantageous positions brought about by social networks.

Enterprises with a low-level social network may have sparse and loose embedded business ecological network structure, weaker connection strength with customers, suppliers, and scientific research institutions. The structural hole occupied by enterprises can also be limited. Although objectively it is not conducive for enterprises to obtain more comprehensive information, enterprises in this context are often forced to weaken or even eliminate their dependence on social networks (Arenius and Clercq, 2005; Ren and Rui, 2014). Instead, they focus on their management to invest and develop human resources and then use human resources more as a means of seizing market opportunities and making strategic adjustments to improve strategic flexibility. Gargiulo and Benassi (2000) suggest that excellent management relies less on external networks, but more on its management capabilities. In this context, when social networks are low-level, enterprises need to rely on HPWS to obtain strategic flexibility.

*Hypothesis 5: Social network moderates the positive relationship between HPWS and strategic flexibility in such a way that the relationship is stronger when the social network is low than when it is high.*

Present research can be extended to a model of the mediated moderation effect. Specifically, strategic flexibility mediates the impact of HPWS on organizational performance; however, this mediating effect depends on the abundance of enterprises’ social networks. Generally speaking, for enterprises with richer social networks, the impact of HPWS on strategic flexibility is weaker, thus the strategic flexibility would less transmit the effect of HPWS on organizational performance. In contrast, for enterprises with fewer social networks, the impact of HPWS on strategic flexibility is stronger, thus the strategic flexibility would more transmit the effect of HPWS on organizational performance.

*Hypothesis 6: Social network moderates the mediation effect of strategic flexibility between HPWS and organizational performance in such a way that the mediation effect is stronger when the social network is low than when it is high.*

**FIGURE 1:**
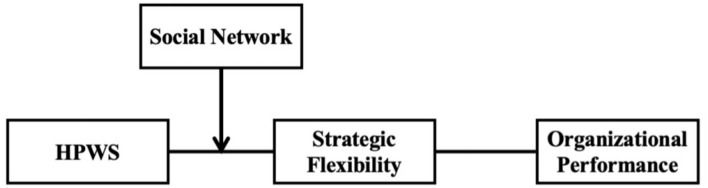
Research model.

The research framework is illustrated in Figure 1.

## Materials and Methods

### Participants

Data collection lasted for 5 months and the participants mainly came from students in an MBA class. They were from various industries and most of them were human resources directors or senior managers of their enterprise. According to the design of research, all related enterprises were over 100 employees because the larger the scale of the enterprise, the more likely it is to implement HPWS. We invited 327 participants to participate in the questionnaire survey. In total, 214 completed the survey and the effective recovery rate was 65.4%. After simple data processing, we found that 83 participants’ enterprises belong to the second industry and that 131 participants’ enterprises belong to the third industry. From the ownership perspective, 92 of the participants’ enterprises belonged to state-owned examples, and 122 of the participants’ enterprises belonged to non-state-owned examples.

### Measures

#### High-Performance Work Systems

We revised the scale based on Miao et al. (2014) to measure HPWS. This scale is composed of 8 dimensions and 33 items, example items are “Compared to skills, companies pay more attention to the basic qualities of employees learning ability when recruiting […]. The training content provided by the company is systematic, such as corporate culture, management/professional skills.” In this research, the Cronbach’s α coefficient of the scale was 0.955.

#### Strategic Flexibility

We revised the scale of strategic flexibility based on the scale of Sanchez (1995, 1997) and Wang (2015). This scale has 2 dimensions: resource flexibility and coordination flexibility, including 9 items, such as “the same resource often has multiple uses” and “enterprises can discover future opportunities and react faster than existing and potential competitors.” In this research, the Cronbach’s α coefficient of the scale was 0.907.

#### Social Network

We revised the scale of social networks based on the scale of Xie et al. (2014). This scale includes 3 dimensions (network scale, network intensity, and network center) and 12 items, example items are “the frequency of cooperation and communication between customers” and “our enterprise is the information distribution center of other enterprises in the corporate network.” In this research, the Cronbach’s α coefficient of the scale was 0.956.

#### Organizational Performance

We used the Chinese version of the scale developed by Xie et al. (2006) for measuring organizational performance. This scale has 2 dimensions: short-term performance and long-term performance, and 12 items, example items are “compared with major competitors, the enterprise’s satisfaction with sales growth rate,” “compared with major competitors, the enterprise’s satisfaction with new product development performance.” In this research, the Cronbach’s α coefficient of the scale was 0.946.

## Results

### Confirmatory Factor Analysis and Common Method Deviation Test

We used AMOS21.0 to conduct the confirmatory factor analysis. The results showed (see Table 1) that comparing with single-factor, two-factor, or three-factor model, the four-factor model fitted the best, which reflected that the variables involved in this research have good discriminative validity.

**TABLE 1:** Confirmatory factor analysis results (*N* = 214).

Model	c2	*df*	c2/*df*	TLI	CFI	RMR	RMSEA
Single-factor	1042.161	90	11.58	0.552	0.616	0.213	0.223
Two-factor	877.833	89	9.863	0.625	0.682	0.200	0.204
Three-factor	479.051	87	5.506	0.809	0.842	0.255	0.145
Four-factor	204.241	84	2.431	0.939	0.952	0.064	0.082

*Single-factor model: Four variables are combined into one factor; two-factor model: Strategic flexibility (SF), social network (SN), and organizational performance (OP) are combined into one factor; three-factor model: SF and SN are combined into one factor.*

To avoid the possible impact of common method deviation, we used Harman’s single factor method to test the homological bias and used SPSS22.0 to conduct the principal component analysis on variables. The result showed that the first factor explains 39.713% of the total variation, which is lower than the standard 40%, so we believe that the common method deviation of the data is within an acceptable range. In addition, all the variables were centered and we found that its tolerance is between 0.55 and 0.99, and VIF (variance inflation factor) is between 1.01 and 1.83, which is lower than the critical value of 10. Therefore, there is no serious multi-collinearity problem in this model.

### Preliminary Analysis

Table 2 provides preliminary analysis results of main variables, including the mean (M), standard deviation (SD), average extraction variance (AVE). The results showed that HPWS is positively correlated with organizational performance (*r* = 0.600, *P* < 0.01), and is positively correlated with strategic flexibility (*r* = 0.719, *P* < 0.01), and strategic flexibility is positively correlated with organizational performance (*r* = 0.701, *P* < 0.01), so the hypothesis 1–3 has been initially verified. The AVE is greater than 0.5 and the square root is greater than the absolute value of the correlation coefficient of each variable, which indicates that the model has good discriminant validity.

**TABLE 2:** Mean value, standard deviation, correlation coefficient, square root of AVE.

Variable	*M*	*SD*	1	2	3	4	α
HPWS	5.13	0.87	0.590				0.955
SF	4.86	1.02	0.719**	0.646			0.907
SN	4.42	1.21	0.260**	0.287**	0.677		0.956
OP	4.52	0.98	0.600**	0.701**	0.280**	0.638	0.946

N = 214, **means p < 0.01. The correlation coefficient is in the lower triangle of the matrix, and the square root of AVE is on the diagonal.

### Hypothesis Testing

We used the SPSS22.0 to conduct the hypothesis testing (mediation and moderation). The independent variable in this research is HPWS, and the dependent variable is organizational performance. Firstly, based on past research experience, we used the enterprise scale, enterprise industry, enterprise ownership, and enterprise location as the control variables (Li and Zhang, 2007). Then, Model 2 showed that HPWS had a significant positive effect on organizational performance (β = 0.608, *p* < 0.001), supporting Hypothesis 1. Model 6 showed that HPWS has a significant positive effect on strategic flexibility (β = 0.723, *p* < 0.001), supporting Hypothesis 2. Model 4 incorporated strategic flexibility into the model and found that HPWS is significantly related to organizational performance (β = 0.221, *p* < 0.001), and strategic flexibility in model M3 is significantly related to organizational performance (β = 0.698, *p* < 0.001), and 0.221 < 0.608, so strategic flexibility mediated the relationship between HPWS and organization performance, supporting the Hypothesis 3 and Hypothesis 4 (see Table 3).

**TABLE 3:** Test of the mediating role of strategic flexibility.

	Organizational performance				Strategic flexibility	
		
	M1	M2	M3	M4	M5	M6
Scale	0.091	0.040	0.073	0.059	0.026	–0.036
Industry	–0.014	0.055	–0.014	0.011	0.000	0.082
Ownership	–0.095	−0.157**	–0.121	−0.137**	0.038	–0.036
Location	0.265***	0.249***	0.128*	0.154**	0.196**	0.177***
HPWS		0.608***		0.221***		0.723***
Strategic flexibility			0.698***	0.535***		
*R* ^2^	0.023	0.387	0.495	0.516	0.026	0.543
△*R*^2^	0.023	0.364**	0.472***	0.129***	0.003**	0.517***
*F*-value	2.277	27.81	42.815	38.800	2.435	51.548

All are standardized coefficients; N = 214, M is model, **means p < 0.01, ***means p < 0.001.

**FIGURE 2:**
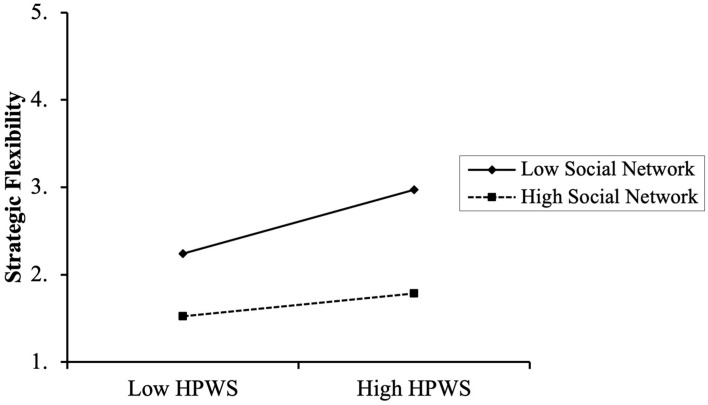
The moderating effect of social networks on the relationship between HPWS and strategic flexibility.

The next step is to test the moderating role of social networks (see Table 4). Based on model M6, the social network was incorporated into model M7, and the result showed that HPWS (β = 0.667, *p* < 0.001) and social network (β = 0.165, *p* < 0.001) had a significant positive effect on strategic flexibility. In model M8, the interaction term between HPWS and social network is significant (β = −0.122, *p* < 0.01), and △*R*^2^ = 0.010 (*p* < 0.001). Thus, hypothesis 5 is supported. As shown in Figure 2, the relationship between HPWS and strategic flexibility is stronger when the social network level is low than when it is high.

**TABLE 4:** Moderating test.

	Strategic flexibility		Organization performance	
		
	M7	M8	M9	M10
Scale	–0.057	–0.052	0.021	0.022
Industry	0.079	0.092	0.053	0.056
Ownership	0.005	0.014	–0.120	–0.118
Location	0.179***	0.163	0.251***	0.246***
HPWS	0.667***	0.660	0.567***	0.562***
Social network	0.165***	0.212	0.146**	0.160**
HPWS*SN		−0.122**		–0.035
*R* ^2^	0.564	0.574	0.419	0.400
△*R*^2^	0.564	0.010***	0.419	0.019**
*F*-value	46.883	41.982	24.860	21.294

All are standardized coefficients; N = 214, M is model, **means p < 0.01, ***means p < 0.001.

Finally, we tested the mediated moderation model based on the method of Muller et al. (2005). The procedures were: (1) regression of dependent variables on independent variables, moderating variables, and interaction terms, the coefficient of the interaction term is significant; (2) regression of the mediating variable to the independent variable, the moderating variable, and the interaction term, the coefficient of the interaction term is significant; and (3) regression of the dependent variable to the independent variable, the moderating variable, the interaction term, and the mediating variable, the mediating variable is significant; if the coefficient of the interaction term in standard (3) is not significant or the significance is reduced, the moderating variable completely or partially moderate the mediating mechanism.

Unfortunately, Model 10 incorporated the interaction term into the model and found that the interaction term was not significant (see Table 4). Therefore, there was no need to do the following test, and hypothesis 6 was rejected.

## Discussion

### Theoretical Implication

This research confirmed that in a dynamic competitive environment, HPWS influence organizational performance through strategic flexibility. This conclusion is consistent with the results of Becker and Huselid (2006); Beltrán-Martín et al. (2008), and Lin and Zhao (2013). It provides a theoretical basis and empirical evidence for further revealing the “black box” between HPWS and organizational performance. Moreover, strategic flexibility only partially mediated the relationship between HPWS and organizational performance, indicating that future research should continue to explore the mechanism of HPWS from other perspectives.

Secondly, this research also tested the moderating role of social networks. Most previous research on this subject paid more attention to the internal situation factors within the enterprise and only a few took the social network into account from the perspective of sociology when exploring the boundary condition of HPWS. Our research combined the social network theory into exploring the impact of relationships formed by enterprises on strategic flexibility. The results showed that social networks negatively moderated the relationship between HPWS and strategic flexibility, which means the richer social network resources an enterprise has, the smaller the impact of HPWS on strategic flexibility will be. In other words, HPWS and the social network is mutual substitution in the process of acquiring strategic flexibility. These conclusions further expand the framework of social network theory.

In addition, this research also investigated whether there is a mediated moderation effect in this model. We found that social networks did not moderate the mediation effect of strategic flexibility and there may be two reasons: firstly, social networks are indispensable for enterprises to survive in business competition, thus social networks are beneficial to the impact of HPWS on organizational performance to a certain extent; secondly, when the enterprise’ social network is excessively rich, the enterprise has too many resources and opportunities, which may cause information confusion for the enterprise. Specifically, enterprises may spend too much time choosing resources and potential opportunities and miss market opportunities. Therefore, we predict that the relationship of HPWS, social network, and organizational performance is not a simple linear relationship, but is likely a quadratic non-linear relationship.

### Practical Implication

This research has at least three practical implications. First, the results showed that the HPWS can improve organizational performance in a dynamic competitive environment. Thus, we recommend that the CEO (Chief Executive Officer) of enterprises construct HPWS. Moreover, it should be noticed that HPWS with “commitment-oriented” and “control-oriented” is more effective for Chinese enterprises. Besides, the CEO should also note that with the overall improvement of the employees’ quality, the composition of HPWS shall gradually transit to practices of “commitment-oriented,” supplemented by practices of “control-oriented.” Secondly, results showed that HPWS influenced organizational performance partly through strategic flexibility. Hence, when enterprises try to improve organizational performance in a dynamic competition environment, the CEO can focus on strategic flexibility and implement HPWS that can promote strategic flexibility. For example, through the implementation of some practices, such as extensive training, employees can master more dimensional knowledge and skills, which can help enterprises in improving their strategic flexibility significantly. Finally, the results showed that HPWS has boundary conditions in promoting strategic flexibility. We found that social networks can negatively moderate the relationship between HPWS and strategic flexibility, which means that the role of HPWS and social networks may conflict with each other in the process of obtaining strategic flexibility. Therefore, the CEO needs to balance the relationship between HPWS and social networks as much as possible.

### Limitation and Future Direction

There are some limitations in this research: firstly, the number of samples is insufficient as we obtained only 214 valid samples. In addition, participants in our survey are people that have close relationships with the author and collaborators. Therefore, the sample was not collected strictly according to the method of a “random sample,” meaning some coefficients may have biased estimates. We suggest that it is necessary to continue to expand the sample size in future research and conduct a “random sample” survey if conditions permit. Secondly, it is difficult to use cross-section data to reveal the causal relationship between variables. Although we tested the common method deviation, we still cannot rule out the possibility of reverse causality. Further research should use longitudinal data to test the hypothesis. Thirdly, we have not made an objective measurement of the social network but only made a subjective evaluation. Future research could explore a new method of overcoming the shortcomings of this subjective evaluation. Finally, we suggest that future research should incorporate a broader sociological perspective into management research.

## Data Availability Statement

The raw data supporting the conclusions of this article will be made available by the authors, without undue reservation.

## Ethics Statement

Written informed consent was obtained from the individual(s) for the publication of any potentially identifiable images or data included in this article.

## Author Contributions

YC and YW: conceptualization, methodology, and writing- original draft preparation. YC, NX, and YW: data curation, software, writing-reviewing, and editing. YW and HC: visualization, investigation, supervision, and validation. All authors contributed to the article and approved the submitted version.

## Conflict of Interest

The authors declare that the research was conducted in the absence of any commercial or financial relationships that could be construed as a potential conflict of interest.

## Publisher’s Note

All claims expressed in this article are solely those of the authors and do not necessarily represent those of their affiliated organizations, or those of the publisher, the editors and the reviewers. Any product that may be evaluated in this article, or claim that may be made by its manufacturer, is not guaranteed or endorsed by the publisher.
